# Etiopathogenesis of Graves-Basedow disease; where we are and where we are going

**DOI:** 10.1186/1756-6614-6-S2-A43

**Published:** 2013-04-05

**Authors:** Janusz Nauman

**Affiliations:** 1 Medical Research Centre of Polish Academy of Sciences, Warsaw, Department of Medicine and Endocrinology, Medical University of Warsaw, Warsaw, Poland

## 

Graves-Basedow disease (GBd) since late fifties has been considered to be autoimmune thyroid disorder where the presence of thyroid stimulating antibodies (TSAb) may lead to hyperthyroidism, thyroid orbitopathy and thyroid dermopathy. During last years it was generally accepted that development of Graves-Basedow disease is dependent on the presence of two or three groups of factors - genetic and environmental or genetic, environmental and endogenous. Familial clustering of GBd, familial presence of thyroid autoantibodies and high concordance in the incidence and clinical course of the disease in monozygotic siblings all suggested that several genes decide about susceptibility to this disorder and likely its phenotypes. On the other hand environmental agents and endogenous factors served as triggers important to the development of the disease, its clinical course and response to given therapy. It was believed that finally, development of GBd (and other autoimmune disorders) is a consequence of lack of balance between the formation of self reactive T cells and central and peripheral tolerance. As a result thyroid was infiltrated by self reactive T cells that in turn affected B cells and led to the production of thyroid stimulating antibodies.

Most recently etiopathogenesis of GBd became further complicated by both clinical and molecular findings. First, GBd can be clinically presented as a hypothyroidism depending on the switch of TSAb production to thyroid blocking antibody (TBAb) production as well as *vice versa*. Some findings suggest that in prone classically hyperthyroid patient the administration of methimazole can provoke switch off of TSAb generation and start of TBAb followed by block of thyroid hormones biosynthesis and quite rapid morphological damage of thyroid cells. It is a pity that in other hypothyroid patient administration of thyroxine can switch on the generation of TSAb and switch off the production of TBAb. Second, it is more and more obvious that autoimmune thyroid disorders and differentiated thyroid cancer (DTC) especially of papillary type (PTC) share some genetic factors (RET/PTC1) and environmental and epidemiological features. In addition presence of GBd disease in patients with DTC leads to increased mortality of cancer patients. Third, microarray DNA analysis of thyroids from GBd patients with severe course of the disease led to the identification of seven additional genes either overexpressed or underexpressed that may be responsible for high level of TSH-R Abs, large goiter size and high free T3/free T4 ratio, features characteristic for patients that poorly respond to anti-thyroid drug therapy. In addition to these clinical observations, the pathogenesis of GBd during the last two-three years was strongly affected by studies suggesting that non sufficient peripheral tolerance was a key to autoimmunity in general, including mechanisms leading to development of Graves-Basedow disease. In a way we returned to Volpe hypothesis developed in the 70-ies of the previous century that pointed out the role of T-suppressor cells. At present the name suppressor cells was postponed and we know that control of self reactive T cells is a function of T regulatory cells (Tregs). It has been proven that block of activation of Tregs both in experimental animals and humans led to the development of generalized fatal autoimmunity. The mechanism of appropriate Tregs activation concerning first signal (MHC-TCR) and especially second costimulatory signals (CD28-B7-1(CD80) or CD28-B7-2 (CD86)) will be presented and their role for the thyroid autoimmunity discussed. The progress in our knowledge concerning autoimmunity as a whole and GBd as a thyroid autoimmune disorder clearly shows that pathogenesis of this disorder is more complex than previously expected and that personalized approach to given patient based on clinical and molecular findings is more important than the hope that single finding (light in corridor) brings a positive solution for all patients with Graves-Basedow disease.

